# DHCLHAM: microbe-drug interaction prediction based on dual-hypergraph contrastive learning framework with hierarchical attention mechanism

**DOI:** 10.3389/fmicb.2025.1657431

**Published:** 2025-10-03

**Authors:** Hailong Hu, Cong Nie

**Affiliations:** ^1^School of Information Engineering, Huzhou University, Huzhou, China; ^2^Zhejiang Key Laboratory of Industrial Solid Waste Thermal Hydrolysis Technology and Intelligent Equipment, Huzhou University, Huzhou, Zhejiang, China; ^3^Zhejiang Key Laboratory of Intelligent Education Technology and Application, Zhejiang Normal University, Jinhua, Zhejiang, China; ^4^Huzhou Key Laboratory of Waters Robotics Technology, Huzhou University, Huzhou, China

**Keywords:** hypergraphs, contrastive learning, microbe-drug interactions, non-linear fusion, network pharmacology

## Abstract

**Introduction:**

Various drugs can markedly disrupt gut microbiota, resulting in a reduction of beneficial microbial populations and precipitating a range of negative clinical consequences. Traditional experimental methods have considerable limitations in clarifying the mechanisms of microbe-drug interactions, thereby necessitating the creation of innovative computational techniques to establish theoretical foundations for personalized and precision medicine. However, the majority of current computational methods rely on graph structures, which inadequately represent the intricate, varied, and heterogeneous interactions among multiple drugs and microbial communities.

**Methods:**

We introduce a hierarchical attention-driven dual-hypergraph contrastive learning framework for predicting microbe-drug interactions. Initially, the original bipartite graph and various similarity data are integrated using nonlinear features by incorporating the functional similarity of medicinal chemical attributes and microbial genomes, alongside computing the Gaussian kernel similarity. Subsequently, a dual network structure comprising K-Nearest Neighbors (KNN) hypergraph and K-means Optimizer (KO) hypergraph is established, employing a hierarchical attention mechanism to facilitate collaborative information aggregation between hyperedges and hypernodes. A contrastive learning approach is implemented to enhance the representation of the heterogeneous hypergraph space, and the prediction scores for microbe-drug interactions are derived by dynamically integrating two-channel embedded features via multi-head attention.

**Results:**

Experiments conducted on various publicly accessible benchmark datasets demonstrate that the DHCLHAM model markedly surpasses the current optimal model in critical metrics, including AUC and AUPR. Particularly on the aBiofilm dataset, the AUC and AUPR attained 98.61% and 98.33%, respectively.

**Discussion:**

A computational framework was developed through multi-dimensional case validation, integrating artificial intelligence and network pharmacology principles, offering a novel paradigm for analyzing microbe-drug interaction mechanisms. The research findings hold significant reference value for optimizing clinical treatment protocols and establish a theoretical foundation to develop precise medication strategies aimed at intestinal flora.

## 1 Introduction

Microorganisms are omnipresent in the human body, encompassing the skin, gastrointestinal tract, and oral cavity, and are essential for sustaining human health (Wu et al., 2024). In homeostatic conditions, the microbial community aids in the body's physiological equilibrium by engaging in nutrient metabolism and influencing immune system development and function. Disruptions in microbial community structure, known as dysbiosis, have been associated with various diseases, including obesity, diabetes, inflammatory bowel disease, and cancer (Goel et al., 2025). Moreover, during therapeutic interventions, drugs may interact with host-associated microorganisms, affecting both drug effectiveness and the composition of the microbial community (Kumbhare et al., 2023). Consequently, a comprehensive understanding of microbe-drug interactions is essential for clarifying disease mechanisms, enhancing therapeutic strategies, and guiding the creation of innovative treatments (Zimmermann et al., 2019). Population-based case-control studies from the United Kingdom and the Netherlands have shown that various commonly prescribed medications, including atypical antipsychotics, non-steroidal anti-inflammatory drugs, and statins, significantly influence the gut microbiota (Liu et al., 2020). Although these clinical studies offer important insights into the impact of pharmacotherapy on the gut microbiome, their scope is inherently restricted and encounters considerable challenges in assessing the complete range of microbe-drug interactions. Fueled by the swift advancement of bioinformatics and computer science, coupled with the growing accessibility of microbial and pharmacological data, computational methodologies have arisen as potent instruments to forecast microbe-drug relationships. These methods efficiently analyze large-scale datasets, identify potential association patterns, and generate insights to guide experimental validation, thereby enhancing traditional research paradigms.

### 1.1 Traditional methods for studying microbe-drug associations

Traditionally, the investigation of microbe-drug relationships has predominantly depended on biological experiments, clinical observations, and empirical treatment methodologies (Long et al., 2022). In laboratory environments, microbial susceptibility and resistance to pharmacological agents are evaluated by culturing microbes *in vitro* and analyzing their growth inhibition or survival in the presence of drugs (Xuan et al., 2024a). Clinical observations concentrate on assessing patient reactions to antimicrobial treatments, especially in instances of particular microbial infections, resulting in the progressive accumulation of empirical treatment protocols. Nonetheless, these conventional methods demonstrate numerous constraints. Laboratory investigations require expensive equipment and specialized knowledge, and *in vitro* conditions frequently do not accurately mimic the complexities of the *in vivo* physiological environment, leading to possible discrepancies between experimental results and real-world situations (Zhou et al., 2024). While clinical observations offer significant practical insights, they necessitate extensive case collection and extended follow-up durations, complicating the timely recognition of generalizable patterns. Furthermore, these methodologies often exhibit an insufficiency of empirical data regarding rare or novel microbe-drug interactions (Rajput et al., 2023).

### 1.2 Computational methods for studying microbe-drug associations

The application of computational methods has significantly advanced the study of microbe-drug associations. Early approaches laid a crucial foundation for this field. For instance, pioneering work like HMDAKATZ (Zhu et al., 2021) successfully demonstrated the utility of heterogeneous networks for deducing potential associations. This model utilized metrics based on node correlations which, while effective, highlighted an opportunity to explore more complex biological representations. Subsequent research introduced graph neural networks, further enhancing predictive capabilities. Models such as GCNMDA (Long et al., 2020a) and EGATMDA (Long et al., 2020b) became influential paradigms. A common practice in these approaches was the use of random negative sampling. This strategy proved effective for model training, yet it does not explicitly differentiate the influence of various negative samples, presenting a potential avenue for refining representation learning and improving prediction accuracy. As the field matured, more sophisticated architectures emerged. The MKGCN model (Yang et al., 2022) sought to extract rich features from complex heterogeneous networks. However, navigating these intricate network structures presents a challenge in capturing the deeper semantic and relational information between nodes, which in turn can influence the model's interpretability. Similarly, methodologies like PCMDA (Gu et al., 2025), which rely on established knowledge graphs, have been instrumental in integrating static biological data. An open research question, however, is how to best incorporate the dynamic nature of microbe-drug associations over time. More recent models have begun to address these challenges. The DHDMP model (Xuan et al., 2024b) made notable strides by incorporating dynamic topological hypergraphs and cross-attention mechanisms. Its comprehensive design, validated on a singular dataset, underscores the trade-off between model complexity and generalizability. Meanwhile, SCSMDA (Tian et al., 2023) introduced structure-enhanced contrastive learning, a powerful technique for improving graph representations. This work brings to light an important consideration: how to augment graph structures without inadvertently introducing noise that could deteriorate the original data's integrity (Hassani and Khasahmadi, 2020). These collective efforts highlight the progress of the field and illuminate the remaining challenges that motivate our present work.

### 1.3 A graph-structured approach for studying microbe-drug associations

Recent advancements in hypergraph structures and graph contrastive learning have provided novel insights to predict microbe-drug associations. Hypergraphs extend traditional graphs by incorporating hyperedges, which can represent higher-order relationships among multiple nodes. This characteristic renders hypergraphs particularly advantageous for numerous bioinformatics applications, such as modeling miRNA-disease associations (Ouyang et al., 2024a). By encompassing these higher-order interactions, hypergraphs can surpass conventional graph-based models in numerous data mining and predictive tasks. The HGCLMDA model (Hu et al., 2023) shows this methodology by forecasting mRNA-drug interactions via the random initialization of hyperedge structures and bipartite graphs, integrating local and global information encoding modules for contrastive learning. A customized contrastive loss function is utilized to refine the embedded representations of mRNAs and drugs, thus augmenting predictive performance. The HyGNN model (Khaled et al., 2023) relies solely on the SMILES strings of drugs to construct a hypergraph and employs a novel attention-based hypergraph edge encoder to learn drug representations, demonstrating superior performance over existing methods on two datasets and highlighting the advantages of hypergraphs in capturing higher-order similarities in drug chemical structures. To predict drug-microbe-disease associations, the MCHNN model (Liu et al., 2023) constructs hypergraph nodes utilizing the characteristics of drugs, microbes, and diseases, employing contrastive learning to enhance the quality of node representations. These achievements highlight the potential of hypergraph structures and contrastive learning in representing biological relationships. However, differences exist in hypergraph construction, which can be categorized into three main types: (1) Direct hypergraph construction utilizing raw linkage data (Ma and Ma, 2022), which is susceptible to overfitting in link prediction tasks; (2) Dynamic hypergraph construction through random initialization (Lu et al., 2024), wherein latent node correlations are refined during training to yield an adaptive hypergraph structure, although this method requires substantial computational resources and experiences diminished interpretability; and (3) Clustering-based hypergraph construction, which employs clustering algorithms on raw data prior to hypergraph modeling (Wu et al., 2020). Our work employs a clustering-based hypergraph construction strategy, leveraging both KNN and KO (Minh et al., 2022) algorithms to create two complementary hypergraph models. The KNN method demonstrates better classification performance for samples with significant class domain crossing and overlapping, which aligns with the characteristics of microorganisms and drugs. Meanwhile, the KO algorithm can more effectively avoid the occurrence of single information and generate more comprehensive hyperedges by dynamically adjusting the cluster centers and optimizing strategies. Considering that microbes and drugs contain various sources of biological information, encompassing functional and structural attributes, we initially calculate multi-view similarity matrices and amalgamate them through non-linear fusion methods. To thoroughly elucidate the intricate structural characteristics of microbial and drug hypergraphs, we compute attention scores for both hyperedges and hypernodes. We additionally compare hypergraphs based on KNN and KO to assess their respective effects in relation to traditional structural enhancement methods. Ultimately, microbe-drug association scores are obtained by synthesizing data from both hypergraphs, using multi-head attention mechanisms to produce microbe-drug embedding features.

This study presents a novel framework that integrates a bi-level attention mechanism with bi-hypergraph contrastive learning to predict microbe-drug interactions. The primary contributions of our research are as follows:

(1) We use original microbe-drug association data, in conjunction with various sources of microbe and drug similarity data, to develop a dual-hypergraph structure employing a combination of KNN and KO clustering algorithms. A non-linear fusion method is used to amalgamate multi-source similarity data, employing normalization and localized similarity calculations to enhance this fusion process.
(2) To manage the high-dimensional data represented in hypergraphs, where hyperedges can include multiple nodes, we develop both a hyperedge-level and a node-level attention mechanism for intra-hypergraph information aggregation. Furthermore, we incorporate bi-hypergraph contrastive learning with a Graph-Transformer to augment and amalgamate dual-view representations.
(3) We establish a computational framework based on network pharmacology principles through multi-dimensional case studies, presenting a novel paradigm to model microbe-drug interaction mechanisms. Our findings offer essential guidance for refining clinical treatment protocols and establish a theoretical basis for the progression of precision medicine strategies aimed at the gut microbiota.

## 2 Materials and methods

**Figure 1:**
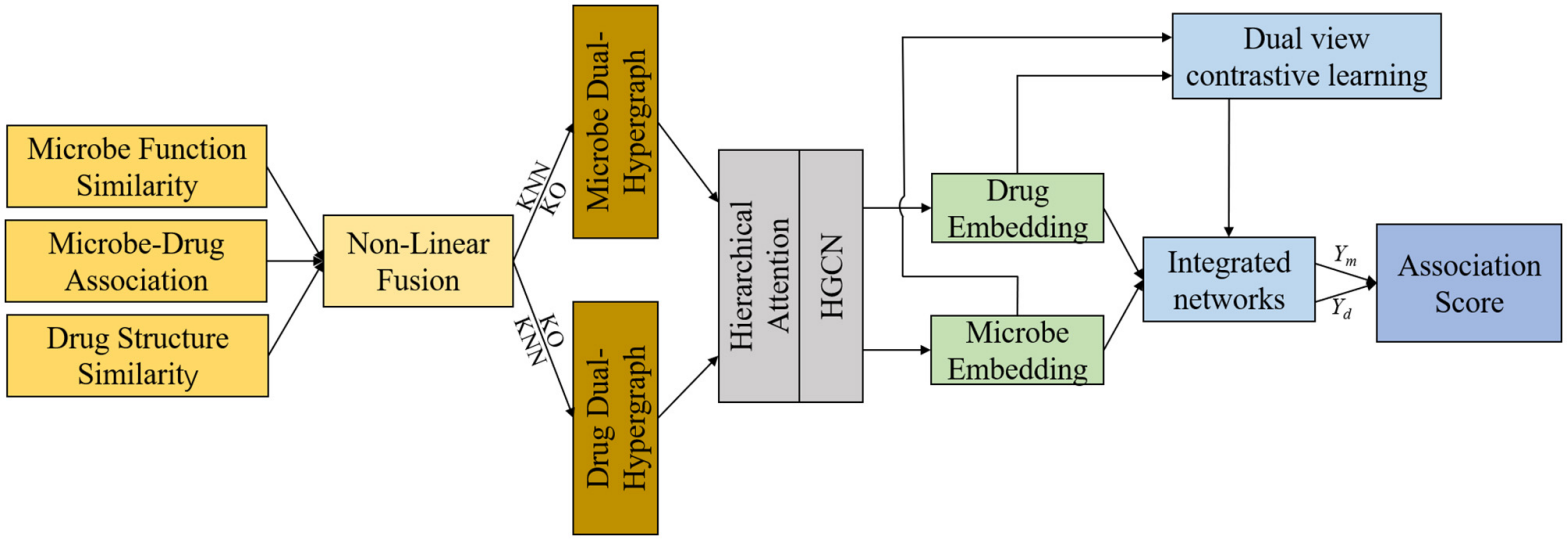
Workflow of the DHCLHAM.

**Figure 2:**
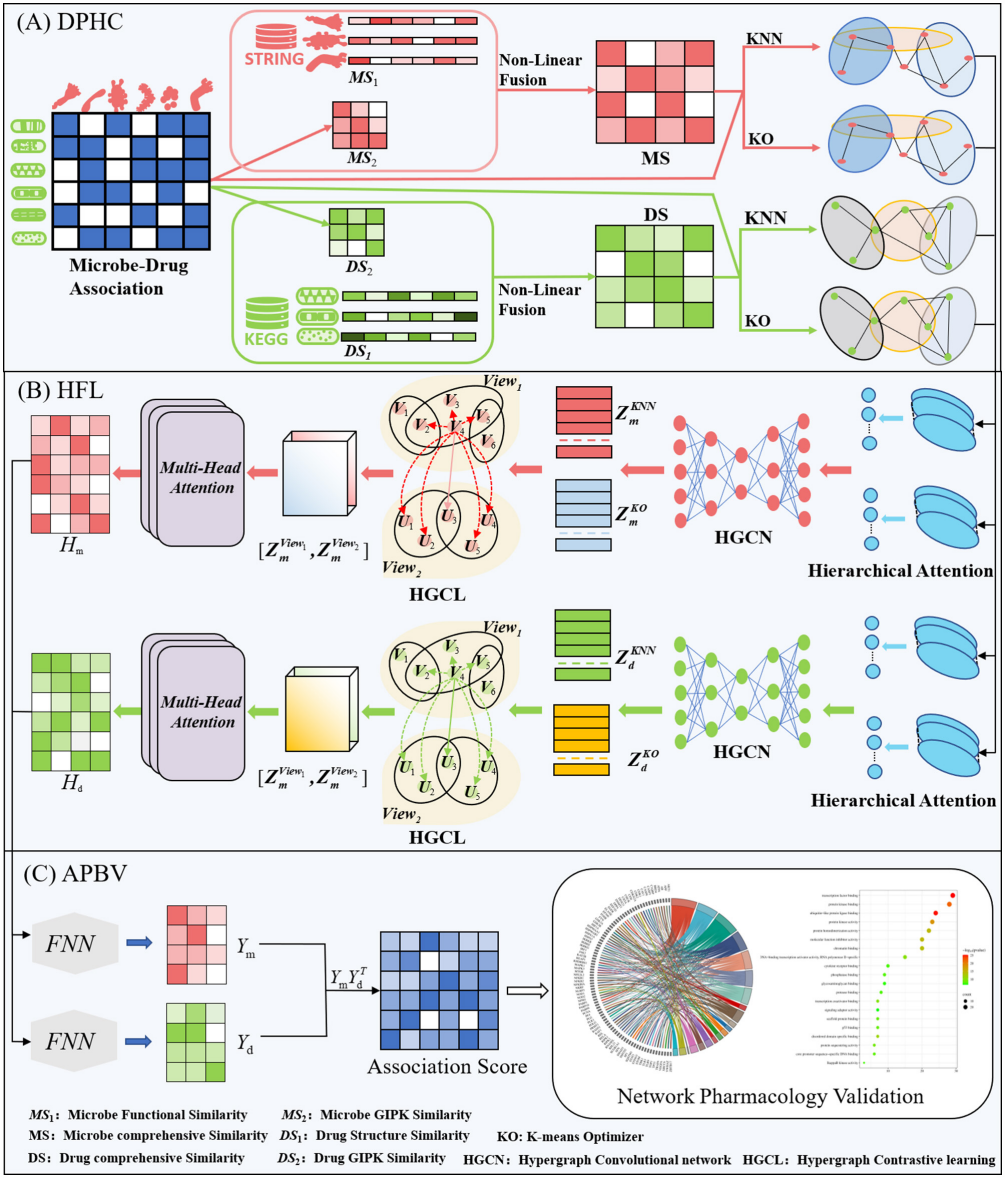
HCLHAM framework diagram **(A)** Data processing and hypergraph construction (DPHC); **(B)** Hierarchical feature learning (HFL); **(C)** Association prediction and biological validation (APBV).

This section offers a succinct summary of the experimental dataset and the essential concepts that form the foundation of our model. (A) Data Processing and Hypergraph Construction (DPHC): Microbe-drug associations are extracted, and functional similarity along with Gaussian kernel similarity is computed for microbes, whereas structural similarity and Gaussian kernel similarity are determined for drugs. The similarity matrices are then combined using a non-linear fusion strategy to produce a comprehensive similarity matrix. Using the fused similarity matrix and the original microbe-drug associations, hypergraphs are constructed through KNN and KO algorithms to delineate the hyperedges. (B) Hierarchical Feature Learning (HFL): A hierarchical attention mechanism is implemented to independently calculate hyperedge-level and node-level attention. Topological characteristics are derived using hypergraph convolutional networks. Moreover, contrastive learning is utilized to augment the discriminative capacity of embeddings across various hypergraph perspectives. A perceptual attention mechanism dynamically integrates multi-view features, while multi-head attention is used to adaptively merge the dual-channel embeddings. (C) Association Prediction and Biological Validation (APBV): The ultimate embedding representations are enhanced through a fully connected layer, and the probabilities of microbe-drug associations are reconstructed via matrix multiplication. To biologically interpret the anticipated interactions, network pharmacology validation is conducted, including target localization, pathway analysis, and functional enrichment analysis. The workflow and detailed process of DHCLHAM are illustrated in Figures 1, 2.

### 2.1 Data collection

Despite the increasing body of research, existing understanding of microbial functions, colonization patterns, and mechanisms of action during pharmacological treatment is still inadequate. In recent years, numerous specialized databases have been established to document microbe-drug interactions, including MDAD (Sun et al., 2018), aBiofilm (Rajput et al., 2018), and DrugVirus (Andersen et al., 2020).

MDAD database, created by Sun et al. in 2018, was assembled through the meticulous curation of experimentally and clinically validated microbe-drug interactions sourced from existing drug databases and scientific literature. It comprises 2470 verified records of microbe-drug associations, involving 1,373 drugs and 173 microbes. The dataset can be accessed publicly at http://chengroup.cu-mt.edu.cn/MDAD.

aBiofilm is a database of anti-biofilm agents that catalogs 1,720 compounds targeting 140 microbial species. The database documents details for each anti-biofilm drug, including molecular structure, drug classification, antimicrobial potency, and citations. The dataset can be accessed publicly at http://bioinfo.imtech.res.in/manojk/abiofilm/.

DrugVirus is a specialized database that records the activity of drugs aimed at human viruses and their interactions. It is intended to enable the investigation and assessment of broad-spectrum antiviral drugs (BSAs), which are agents that suppress various human viruses, along with categories of drugs that include BSAs. The database can be accessed at https://drug-virus.info/.

Table 1 presents statistical information regarding the three datasets, their densities are 1.04%, 1.19%, and 5.61%, respectively.

**Table 1:** Statistical information on microbial and pharmaceutical datasets.

**Datasets**	**Microbes**	**Drugs**	**Associations**	**Densities**
MDAD	173	1,373	2,470	1.04%
aBiofilm	140	1,720	2,884	1.19%
DrugVirus	95	175	933	5.61%

### 2.2 Data processing and hypergraph construction

#### 2.2.1 Microbe similarity network construction

This study evaluates microbial similarity through two methodologies. The initial category of microbial similarity is functional similarity, determined through the Kamneva (Kashyap et al., 2017) algorithm. Assuming the existence of two microbes, *m*_*i*_ and *m*_*j*_, their functional similarity can be represented by *MS*_1_(*m*_i_, *m*_j_). However, many microbes do not have similarity scores in *MS*_1_, and obviously that *MS*_1_ is sparse, and additional similarity information must be obtained to uncover more valuable microbial insights. The second microbial similarity is Gaussian interaction profile kernel similarity, which posits that analogous microbes exhibit comparable functions, leading to similar interaction profiles. The Gaussian kernel similarity effectively harnesses the interaction information among nodes within the network. Consequently, it offers a robust approach to measure the similarity among nodes. Specifically, in the original matrix *A*, microbes *m*_*i*_ and *m*_*j*_ can be represented as rows *i* and *j* in *A*. The Gaussian kernel similarity *MS*_2_ (*m*_*i*_, *m*_*j*_) of microbes *m*_*i*_ and *m*_*j*_ is defined in Equation 1.


(1) $$\begin{array}{l} MS_{2}\left(m_{i},m_{j}\right)=\exp\left(-\eta_{m}∥A\left(m_{i},▪\right)-A\left(m_{j},▪\right){∥}^{2}\right) \end{array}$$

Where $\eta_{m}={\eta^{\prime }}_{m}/\left(\frac{1}{N_{m}}\overset{N_{m}}{\underset{i=1}{\sum }}{∥A\left(m_{i},▪\right)∥}^{2}\right)$, ${\eta^{\prime }}_{m}$ is the raw bandwidth, always set to 1.

#### 2.2.2 Drug similarity network construction

This model assesses drug similarity through two methods. The initial aspect is structural similarity, computed via the SIMCOMP2 algorithm (Hattori et al., 2010). Based on chemical structure information, we measure drug similarity by mapping dataset drugs to those in KEGG and obtaining structure similarity with a custom cut-off score of 0.5. For two drugs *d*_*i*_ and *d*_*j*_, their structural similarity is expressed as *DS*_1_(*d*_*i*_, *d*_*j*_). However, many drugs lack a similarity score in *DS*_1_, and evidently that *DS*_1_ is sparse. To derive more comprehensive similarity information, additional data sources must be identified. The second type of drug similarity is the Gaussian interaction profile kernel similarity (*DS*_2_), computed similarly to the microbial Gaussian interaction profile kernel similarity.

#### 2.2.3 Non-linear fusion of microbe and drug similarity networks

**Figure 3:**
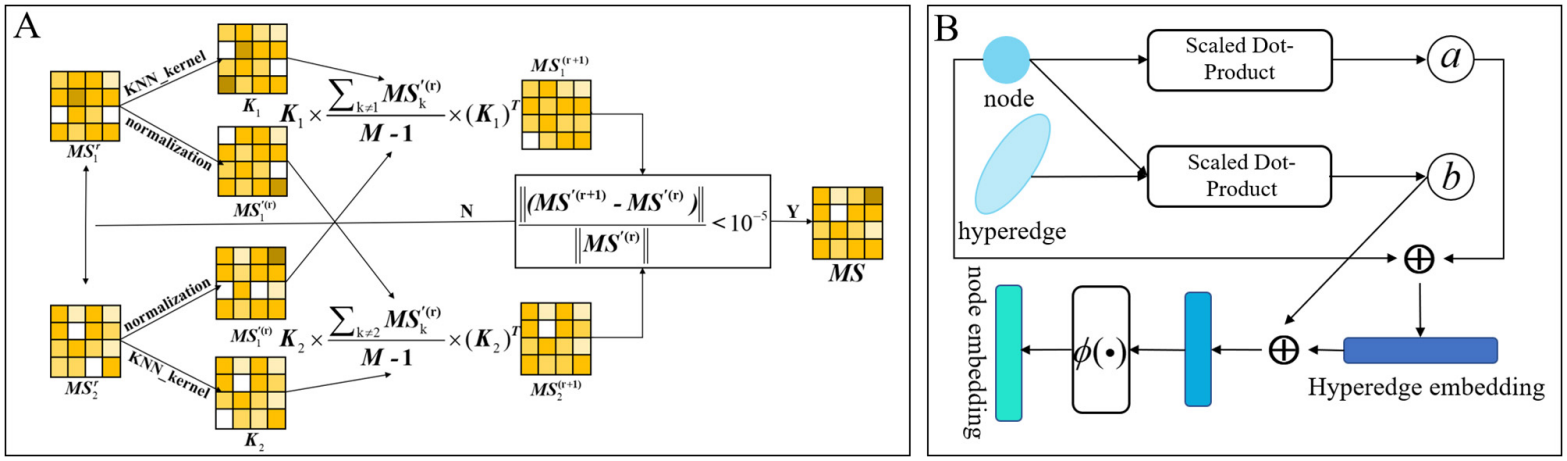
**(A)** Calculated graph of non-linear fusion of multiple similarities. **(B)** Diagram of the process of calculating hierarchical attention in hypergraphs.

We use microbes as an example. Following the calculation of microbial functional similarity and the kernel similarity of Gaussian interaction profiles, we integrated the two metrics. Incorporating various similarity measures not only mitigates data bias but also produces a more precise and rational aggregated similarity for microbes and drugs. Nevertheless, basic linear similarity combination techniques frequently prove inadequate for integrating multiple biological similarities. Conventional linear fusion techniques often depend on overly simplistic approaches for integrating multi-view similarity information (Ouyang et al., 2024b), such as substituting absent similarity values with an alternative similarity type or directly averaging various types of similarities. This method fails to adequately represent the intricate non-linear relationships among various similarity types, which may result in information loss or inferior fusion outcomes. Conversely, non-linear fusion can dynamically encapsulate the non-linear interactions among various similarity networks via a sophisticated iterative computation process, thereby allowing for a more exhaustive investigation of the profound information concealed within multi-source data and aiding in the development of a more precise and comprehensive integrated similarity network. Consequently, we used non-linear fusion in this model. The non-linear fusion process involves first calculating the normalized weights and local relationships for each similarity matrix. Subsequently, these normalized weights and local relationships from different similarity matrices undergo iterative computation until they fall below our specified threshold. The flow is shown in Figure 3A. The combined microbial similarity was indicated as *MS*. The normalized weights were calculated and specified as indicated in Equation 2.


(2) $$\begin{array}{l} MS_{t}^{\prime }\left(m_{i},m_{j}\right)=\{\begin{array}{ll} MS_{t}\left(m_{i},m_{j}\right)/\left(2\sum_{k\neq i}^{N_{m}}MS_{t}\left(m_{i},m_{k}\right)\right), & j\neq i\\ 1/2, & j=i \end{array} \end{array}$$

In each similarity network, the KNN algorithm is employed to assess local relationships. For each microbial node, the algorithm identifies its k nearest neighbors, sums the similarities to these neighbors, and normalizes each neighbor's similarity by dividing it by the total sum, thereby generating a KNN similarity matrix as described in Equation 3.


(3) $$\begin{array}{l} K_{t}\left(m_{i},m_{j}\right)=\{\begin{array}{ll} MS_{t}\left(m_{i},m_{j}\right)/\left(\sum_{k\in N_{i}}MS_{t}\left(m_{i},m_{k}\right)\right), & j\in N_{i}\\ 0, & \text{otherwise} \end{array} \end{array}$$

Where *N*_*i*_ is a set of *k* nearest neighbors of node *m*_*i*_ in the microbial similarity network. *K*_*t*_ denotes the local affinity kernel of the *t*th data type, and after many experiments, the neighbor parameter of KNN is taken as *N*_*m*/_10. Finally, this model iteratively revises the similarity matrix for each data type according to the procedure outlined in Equation 4.


(4) $$\begin{array}{l} MS_{t}^{\prime \left(r+1\right)}=K_{t}\times \frac{\sum_{k\neq t}MS_{t}^{\prime \left(r+1\right)}}{M-1}\times {\left(K_{t}\right)}^{T} \end{array}$$

Where *t* = 1, 2, ⋯ , *M, M* is the total number of data types. $MS_{t}^{\prime \left(r+1\right)}$ is the state matrix of the *t*th data type after the *r*th iteration.

In this model, the iteration stops when $MS_{t}^{\prime \left(r+1\right)}$ reaches the convergence criterion, which is defined as the relative change $∥MS_{t}^{\prime \left(r+1\right)}-MS_{t}^{\prime \left(r\right)}∥/∥MS_{t}^{\prime \left(r\right)}∥$ is less than 10^−5^. After iterative updating, the final integrated similarity network *MS* can be obtained defined as shown in Equation 5.


(5) $$\begin{array}{l} MS=\frac{MS_{1}^{\prime \left(r\right)}+MS_{2}^{\prime \left(r\right)}+\cdots +MS_{M}^{\prime \left(r\right)}}{M} \end{array}$$

However, the resulting similarity matrix is not symmetric; thus, $MS_{final}=\left(MS+{\left(MS\right)}^{T}\right)/2$ serves as the final microbial similarity matrix. The drug integrated similarity network is equivalent to the non-linear fusion computation of the microbial integrated similarity matrix previously described.

#### 2.2.4 Dual views hypergraph construction

Traditional graph structures inadequately represent the intricate entity interactions inherent in microbe-drug association prediction (Mei et al., 2024). The relationship between microbes and drugs is not merely a straightforward pairwise interaction. Furthermore, drugs targeting the same microorganism frequently possess analogous characteristics, akin to the similarities observed among microbes. By linking drugs with analogous characteristics through hyperedges, the complete network can be depicted as a higher-order graph. Creating hyperedges to investigate higher-order relationships among nodes enables a more thorough examination of intricate interactions within biological systems. Consequently, we utilize the hypergraph structure as an intermediary framework for the transmission of microbial and drug information, enabling the global dissemination of higher-order information between microbial and drug nodes. This model uses a weighted hypergraph *G* = (*V, E, W*) to represent microbe- and drug-related hyperedges. Here, *V* constitutes the vertex set of microbe and drug nodes, *E* is the set of hyperedges, and *W* represents a diagonal weight matrix. The original association matrix and the fused similarity matrix are concatenated to form node features for microbes and drugs. This model employs KNN and KO algorithms to construct hypergraphs for microbes and drugs based on the concatenated features. In the KNN algorithm, we initially compute the nearest *k* neighbors of each microorganism using Euclidean distance, and subsequently identify a subset, referred to as a hyperedge, from the *k* neighbors. Hypergraph structure is called *View*_1_ is designated in this paper. In addition, we employ the KO algorithm to select cluster centers *c* by constructing a fitness function based on the Euclidean distances between each microbe and these centers. By generating high-quality search spaces and directions through centroid-based methods, the algorithm optimizes the positions of cluster centers, thereby enhancing clustering accuracy. Microbe with closer distances are thereby grouped into subsets, referred to as hyperedges; this hypergraph structure is denoted as *View*_2_ in this paper. After many iterations, the clustering center no longer changes. At this point, the obtained relationship matrix of hypergraph hypernodes and hyperedges can be expressed as *H* ∈ *R*^*V*×*E*^. In particular, in *View*_1_ hypergraph, the number of KNN constructed hyperedge of hypergraph is the number of nodes, and in *View*_2_ hypergraph, the number of KO constructed hyperedge of hypergraph is the number of cluster centers. Here, the hypergraph association matrix is represented as *H*. When node *v* belongs to the hypergraph *e, H* (*v, e*) = 1; otherwise, *H* (*v, e*) = 0.

### 2.3 Hierarchical feature learning

#### 2.3.1 Hierarchical attention mechanisms

Graph attention and hypergraph attention mechanisms are intended to elucidate the complex relationships among nodes and edges (or hyperedges) within graph and hypergraph frameworks, respectively, to ascertain the relative significance of each edge (or hyperedge) to a node (Lee and Chae, 2024). This arises from the adjacency matrix of a hypergraph, which includes both nodes and hyperedges, with each hyperedge capable of encompassing multiple nodes. From a specific viewpoint, a singular hyperedge may theoretically encompass all nodes within the graph. Hypergraph attention utilizes the initial node features as input, modifies the representations of the hyperedges, and then consolidates information from these revised hyperedges to enhance the representations of the associated nodes. Acknowledging that some nodes may possess more significant or informative attributes than others, we present a hierarchical attention mechanism. This mechanism initially calculates attention scores at the hyperedge level and then incorporates these scores in the computation of node-level attention. This method allows the model to better maintain the global structural attributes of the hypergraph by adjusting the significance attributed to hyperedges. The proposed model integrates attention mechanisms at both the hyperedge and node levels. Figure 3B schematically shows the dynamic flow of information and the feature enhancement process between nodes and hyperedges within the hypergraph. The attention mechanism first calculate the hyperedge-level attention, then calculate the node-level attention, and finally update the features of the nodes. Here, the collection of all nodes associated with a hyperedge *e*_*i*_ is defined as *y*_*i*_, while the collection of hyperedges linked to a node *n*_*i*_ is denoted as ρ_*i*_.

#### 2.3.2 Constructing hyperedge-level attention

The model encapsulates information regarding all nodes associated with each hyperedge. The hyperedge-level attention identifies the differing significance of all nodes *n*_*p*_ ∈ *y*_*i*_ associated with a specific hyperedge *e*_*j*_. The attention score *a*_*ji*_ of a node *n*_*i*_ to a hyperedge *e*_*j*_ is described in Equation 6.


(6) $$\begin{array}{l} a_{ji}=\frac{S\left(w_{1}n_{i},u\right)}{\sum_{p\in y_{j}}S\left(w_{1}n_{p},u\right)} \end{array}$$

Where *w*_1_ is the learnable parameter matrix, *u* is the trainable weight vector. We explored effectively address the issue of numerical instability in high-dimensional hypergraph features and enable feature transformation using learnable matrices, the similarity function *S*(▪) is defined as scaled dot-product attention, denoted as $S\left(q,k\right)=q^{T}k/\sqrt{D}$, where *D* is the feature dimension. Unlike cosine similarity, which enforces vector length normalization and discards magnitude information, scaled dot-product attention directly utilizes the original feature magnitudes to preserve the intensity of node features, which is critical for capturing absolute importance in hypergraph structures. Furthermore, the *q*^*T*^*k* computation of scaled dot-product attention is inherently compatible with matrixized operations, enabling efficient GPU acceleration. This significantly reduces computational overhead compared to non-linear metrics like Euclidean distance, making it suitable for end-to-end training on large-scale hypergraphs with complex high-order relationships.

#### 2.3.3 Constructing node-level attention

The revised hyperedge representation is employed to derive the node representation. The node attention assesses the varying significance of distinct hyperedges, and the attention score *b*_*ij*_ of hyperedge *e*_*j*_ to node *n*_*i*_ is defined as shown in Equation 7.


(7) $$\begin{array}{l} b_{ij}=\frac{S\left(w_{2}e_{j},w_{3}n_{i}\right)}{\sum_{p\in \rho_{i}}S\left(w_{2}e_{p},w_{3}n_{i}\right)} \end{array}$$

Where *w*_2_ and *w*_3_ are learnable parameter matrices.

Finally the hyperedge representation *e*_*j*_ = ∑_*i*∈*y*_*i*__*a*_*ji*_*w*_1_*n*_*i*_ and the updated node representation *Z*_*i*_ = ϕ(∑_*j*∈ρ_*i*__*b*_*ij*_*w*_2_*e*_*j*_), are derived by aggregating their neighbor information, here ϕ(▪) consists of two layers of MLPs and an ELU activation function.

#### 2.3.4 Hypergraph convolutional networks

We employ hierarchical attention to generate information-rich, context-aware node embeddings, which are subsequently fed into the hypergraph convolutional network as high-quality inputs to learn the global topological structure of the entire hypergraph. The hypergraph convolutional network (HGCN), which employs updated nodes for spectral convolution, effectively encodes higher-order relationships within the hypergraph structure (Yang et al., 2024). Feature transformation and hypergraph aggregation facilitate the capture of deeper features, enhanced expressiveness, and improved generalization. Following the adjustment of the nodes, the update formula is delineated in Equation 8, predicated on the weights of the matrix and hyperedges.


(8) $$\begin{array}{l} X^{l+1}=\sigma \left(D_{v}^{-1/2}HWD_{e}^{-1}H^{T}D_{v}^{-1/2}X^{l}\theta^{l}\right) \end{array}$$

Where *X*^*l*^ is the aggregated information at layer *l*,*X*^0^ = *X*. θ^*l*^ is the learnable weight matrix, and σ(▪) is the non-linear activation function. *D*_*e*_ is the degree matrix of the hyperedge, defined as *d*(*e*) = ∑_*v*∈*V*_ *H*(*v, e*). *D*_*v*_ is the degree matrix of the node, defined as *d*(*v*) = ∑_*e*∈*E*_ *w*(*e*)*H*(*v, e*). After being updated by HGCN, the embedding representations of microbes and drugs are denoted as $Z_{m}^{HGCN}$ and $Z_{d}^{HGCN}$.

#### 2.3.5 Dual views contrastive learning

To tackle the issues of data sparsity and intricate relationships present in real-world hypergraph structures, contrastive learning has become a prevalent approach in recent years. Simultaneously, various studies have combined contrastive learning with graph-structured data to improve the quality of embedding representations. Current GCL-based feature extraction techniques can be classified into two primary categories: structural augmentation and feature augmentation (Hu et al., 2023). Structural augmentation systematically eliminates nodes or edges from the graph to produce a modified structure, which is subsequently processed through an encoder to yield contrastive representations. Conversely, feature augmentation incorporates random noise into node embeddings to generate alternative perspectives for contrastive learning. However, both strategies demonstrate significant shortcomings. Structural augmentation may compromise the intrinsic properties of the original graph by indiscriminately removing nodes or edges, thereby jeopardizing the graph's semantic integrity. Likewise, feature augmentation uniformly applies noise to all nodes, disregarding the distinct attributes and contextual information of each node. This study employs a dual-view contrastive learning framework to address these issues. The dual hypergraph structure effectively addresses both issues: maintaining the fundamental structure of the input graph while reducing the potential for node feature deterioration caused by indiscriminate noise introduction. The suggested dual-view hypergraph contrastive learning approach guarantees the coherence of embeddings for identical nodes across various views and the differentiation of embeddings among disparate nodes. A contrastive objective function is utilized, using the two previously established hypergraph structures. This objective ensures that the encoded representations of each node in the two views are aligned and remain discriminative in relation to the representations of other nodes. That is, for each node *v*, its embedding *v*_*i*_ generated in one view is designated as an anchor point, and the embedding generated in the other view is denoted as *u*_*i*_, such that different embeddings *v*_*i*_ and *u*_*i*_ of the same node in different two views form positive sample pairs. Embeddings *v*_*k*_ and *u*_*k*_(*k* ≠ *i*) of the other nodes are considered as negative sample pairs, where *v*_*k*_ forms an inner view negative sample pair with anchor point *v*_*i*_ and *u*_*k*_ forms a cross-view negative sample pair with anchor point *v*_*i*_. In contrastive learning, we employ InfoNCE (Ouyang et al., 2024a) to guide the model in learning the similarities and differences between data samples. The sample pairs for each positive example are defined in Equation 9.


(9) $$\begin{array}{l} L_{cl}\left(v_{i},u_{i}\right)=\\ -\log\frac{e^{sim\left(g\left(v_{i}\right),g\left(u_{i}\right)\right)/\tau }}{\begin{array}{c} e^{sim\left(g\left(v_{i}\right),g\left(u_{i}\right)\right)/\tau }+\sum_{k\neq i}e^{sim\left(g\left(v_{i}\right),g\left(v_{k}\right)\right)/\tau }\\ +\sum_{k\neq i}e^{sim\left(g\left(v_{i}\right),g\left(u_{k}\right)\right)/\tau } \end{array}} \end{array}$$

Where *sim*(▪) is the cosine similarity function and *g*(▪) a two-layer neural network projection head utilized to augment the informational capacity of the nodes, τ is temperature control parameter.

The hypergraph contrastive loss functions of microbes and drugs are defined Equation 10.


(10) $$\begin{array}{l} \{\begin{array}{l} L_{View_{k}}^{\left(m\right)}\left(v_{i},u_{i}\right)=\\ -\overset{N_{m}}{\underset{i=1}{\sum }}\log\frac{e^{sim\left(g\left(v_{i}\right),g\left(u_{i}\right)\right)/\tau }}{e^{sim\left(g\left(v_{i}\right),g\left(u_{i}\right)\right)/\tau }+\sum_{k\neq i}e^{sim\left(g\left(v_{i}\right),g\left(v_{k}\right)\right)/\tau }+\sum_{k\neq i}e^{sim\left(g\left(v_{i}\right),g\left(u_{k}\right)\right)/\tau }}\\ L_{View_{k}}^{\left(d\right)}\left(v_{i},u_{i}\right)=\\ -\overset{N_{d}}{\underset{i=1}{\sum }}\log\frac{e^{sim\left(g\left(v_{i}\right),g\left(u_{i}\right)\right)/\tau }}{e^{sim\left(g\left(v_{i}\right),g\left(u_{i}\right)\right)/\tau }+\sum_{k\neq i}e^{sim\left(g\left(v_{i}\right),g\left(v_{k}\right)\right)/\tau }+\sum_{k\neq i}e^{sim\left(g\left(v_{i}\right),g\left(u_{k}\right)\right)/\tau }} \end{array} \end{array}$$

Where *N*_*m*_ and *N*_*d*_ denote the number of nodes for microbes and drugs, respectively, and *View*_*k*_ (*k* = 1, 2) represents the *View*_1_ and *View*_2_ hypergraphs. The perspectives on microbes and drugs are symmetrical in the two distinct *View*_1_ and *View*_2_ hypergraph representations of the nodes. Therefore, the final overall contrastive loss functions for microbes and drugs is denoted in Equation 11.


(11) $$\begin{array}{l} \{\begin{array}{c} L_{cl}^{\left(m\right)}=L_{View_{1}}^{\left(m\right)}\left(v_{i},u_{i}\right)+L_{View_{2}}^{\left(m\right)}\left(u_{i},v_{i}\right)\\ L_{cl}^{\left(d\right)}=L_{View_{1}}^{\left(d\right)}\left(v_{i},u_{i}\right)+L_{View_{2}}^{\left(d\right)}\left(u_{i},v_{i}\right) \end{array} \end{array}$$

#### 2.3.6 Integrated networks

Subsequent to employing contrastive learning, we trained the contrast loss from two distinct perspectives. Afterwards, we aim to amalgamate the two perspectives to create a more comprehensive embedding feature vector. Initially, due to the inherent differences between *View*_1_ and *View*_2_ views, the variation in hyperedges influences the microbial and drug embedding feature vectors, resulting in inconsistent preferences between the two views, thereby affecting the final prediction of microbe-drug associations differently. A global average pooling layer, followed by a fully connected neural network, is employed to calculate the weights for each view. The embedding representation is ultimately integrated with the attention weights, defined here using microbes as an example in Equation 12:


(12) $$\begin{array}{l} \{\begin{array}{c} Z_{m}^{View_{1}}=ReLU\left(FNN\left(GAP\left(Z_{m}^{HGCN\left(View_{1}\right)}\right)\right)▪Z_{m}^{HGCN\left(View_{1}\right)}\right)\\ Z_{m}^{View_{2}}=ReLU\left(FNN\left(GAP\left(Z_{m}^{HGCN\left(View_{2}\right)}\right)\right)▪Z_{m}^{HGCN\left(View_{2}\right)}\right) \end{array} \end{array}$$

Where *GAP*(▪) is a global average pooling layer, *FNN*(▪) is a two-layer fully connected neural network where the non-linear activation functions of the two layers are *ReLU*() and Sigmoid() functions, and $Z_{m}^{HGCN\left(▪\right)}$ represents the embeddings of the microbes for the *View*_1_ and *View*_2_ views. The final microbial embedding representation with attentional weights is obtained $Z_{m}=\left[Z_{m}^{View_{1}},Z_{m}^{View_{2}}\right]$. Similarly, the attention weight embedding of the drug can be obtained $Z_{d}=\left[Z_{d}^{View_{1}},Z_{d}^{View_{2}}\right]$.

Utilizing attentional embedding, we acquire the embedding information of the two hypergraph structures. Drawing from Graph-Transformer (Ma et al., 2023), we present a multi-head attention mechanism to synthesize various perspectives of microbes and drugs. In summary, using microbes as an example, the multi-head attention mechanism extracts feature from various subspaces in each self-attention layer, which are subsequently combined to derive the features of the microbes. The computed $Z_{m}^{View_{1}}$ and $Z_{m}^{View_{2}}$ are initially concatenated to produce a composite representation of the microbes $Z_{m}=\left[Z_{m}^{View_{1}},Z_{m}^{View_{2}}\right]$ and eventually $Z_{m}=\left[Z_{m}^{1},Z_{m}^{2}\right]$. Utilizing the Transformer's framework, we project the microbial final representation data onto three fundamental components: the microbial query matrix $Q_{m}=W_{q}Z_{m}=\left[q_{m}^{1},q_{m}^{2}\right]$, the microbial key matrix $K_{m}=W_{k}Z_{m}=\left[k_{m}^{1},k_{m}^{2}\right]$, and the microbial value matrix $V_{m}=W_{v}Z_{m}=\left[v_{m}^{1},v_{m}^{2}\right]$, via three projection weight matrices *W*_*q*_, *W*_*k*_, and *W*_*v*_. Meanwhile, based on the *q*_*m*_ and *k*_*m*_ calculated above and on Scaled Dot-Product Attention, the inter-view attention matrix *A*_*m*_ can be defined as Equation 13.


(13) $$\begin{array}{l} A_{m}\left(i,j\right)=\frac{\exp\left[q_{m}^{i}▪{\left(k_{m}^{j}\right)}^{T}/\sqrt{d_{f}}\right]}{\sum_{j=1}^{2}\exp\left[q_{m}^{i}▪{\left(k_{m}^{j}\right)}^{T}/\sqrt{d_{f}}\right]} \end{array}$$

Where *j* represents *View*_1_ and *View*_2_, *A*_*m*_(*i, j*) denotes the attention of the *i*th view to the *j*th view of the current microbe, and *d*_*f*_ is the dimension of the microbe's embedded representation, so $A_{m}\in R^{2\times 2}$ represents the inter-view attention matrix in both views of the microbe. The attention matrix corresponds directly to the quantity of microbial nodes. The interactions among the various views can be emphasized based on the attention scores of the two perspectives. To enhance the capture of richer feature representations and to improve the robustness and generalization of the model learning process, a self-attention mechanism is implemented as multi-head attention. The definition is specified in Equation 14.


(14) $$\begin{array}{l} V\text{\_}ave_{m}=\frac{1}{N}\overset{N}{\underset{p=1}{\sum }}{\left({\left(A_{m}▪V_{m}^{T}\right)}^{T}\right)}^{p} \end{array}$$

Where *N* denotes the quantity of multi-heads, determined subsequent to the parametric analysis in the experiment. Ultimately, we encode the feature vector embedding derived from the multi-head attention using a two-layer feedforward network to achieve the final embedding representation *h*_*m*_ = *W*_*h*_·Vec(*V*_*ave*_*m*_), where *W*_*h*_ is a parameter in the feedforward network, and *Vec*(▪) represents the vectorization operation of row concatenation, i.e., multiple vectors are concatenated by rows to form a long vector. Then, for *N*_*m*_ microbes, the embedding matrix can be expressed as *H*_*m*_ = [*h*_1_, *h*_2_, ⋯ , *h*_*N*_*m*__]. Similarly, the embedding matrix *H*_*d*_ = [*h*_1_, *h*_2_, ⋯ , *h*_*N*_*d*__] for drugs can be obtained.

### 2.4 Association prediction

In the concluding phase of score prediction, we employ the final embedding representations *H*_*m*_ and *H*_*d*_ of microbes and drugs acquired previously. After that, we derive the embedding matrices for both microbes *Y*_*m*_ = *FNN*(*H*_*m*_) and drugs *Y*_*d*_ = *FNN*(*H*_*d*_) using a fully connected neural network (FNN). Subsequently, the reconstructed correlation matrices are generated through the matrix multiplication of the two features, as delineated in Equation 15.


(15) $$\begin{array}{l} As=Y_{m}Y_{d}^{T} \end{array}$$

### 2.5 Loss function

The initial association matrix of microbes and drugs is sparse, with sparsity levels of 1.04%, 1.19%, and 5.61% for the three datasets, respectively. The quantity of unobserved terms significantly exceeds that of observed terms, creating an imbalance that impacts model training. To address this issue, we employ a trade-off parameter α to equilibrate the observed and unobserved terms, and the model's objective function is delineated as presented in Equation 16.


(16) $$\begin{array}{l} L_{RE}=\frac{\left(1-\alpha \right)}{2}∥P_{\Omega }\left(A-As\right){∥}_{F}^{2}+\frac{\alpha }{2}∥P_{\bar{\Omega }}\left(A-As\right){∥}_{F}^{2} \end{array}$$

Where Ω and $\bar{\Omega }$ are used for observed and unobserved entries, respectively, *A* is the true value matrix, *As* is the prediction matrix, and || ||_*F*_ is the Frobenius norm.

The comprehensive loss function for model optimization comprises the reconstruction loss and the comparative loss between microbes and drugs, as delineated in Equation 17.


(17) $$\begin{array}{l} L=L_{RE}+\lambda L_{cl}^{\left(m\right)}+\gamma L_{cl}^{\left(d\right)} \end{array}$$

Where λ and γ are control coefficients for regulating the comparative loss of microbes and drugs, and considering the experimental complexity, λ and γ are uniformly set to 1.

## 3 Experiments and results

This section provides a thorough experimental assessment of DHCLHAM. We evaluate DHCLHAM against multiple baseline methods to illustrate its performance. Visualization experiments underscore the distinguishing ability of the microbial and drug node embeddings produced by DHCLHAM. Ablation studies are performed to evaluate the contribution of each module in the model. Ultimately, a parameter sensitivity analysis is conducted to facilitate model refinement and optimization.

It is worth noting that, DHCLHAM uses the Adam optimizer for training and applies a grid search strategy to tune its parameters. Ultimately, the learning rate is set to 0.0001, and the trade-off parameter α is 0.11. In the biological correlation encoding component, the dimension is set to 256. During training, DHCLHAM achieves the highest evaluation value at 400 epochs. All experiments are conducted on a desktop with an Intel Core i5-13400F CPU and an NVIDIA RTX4060Ti 8GB GPU. The software environment includes PyCharm 2024.1, Python v3.9.0, Pytorch v2.1.0, NumPy v1.26.0, scikit-learn v1.5.2, and scipy v1.13.1.

### 3.1 Efficiency analysis

As depicted in Figure 2, the training process of DHCLHAM consists of three main steps constructing an integrated similarity network and dual hypergraphs, implementing a dual-level hypergraph attention mechanism and dual hypergraph contrastive learning, and finally integrating the networks via an integrated network.

In the first step, given *m* microbes and *n* drugs, DHCLHAM calculates the similarities among microbes and among drugs, respectively. This process has a time complexity of *O*(*m*^2^) + *O*(*n*^2^). Subsequently, the model employs non-linear fusion to create integrated similarity networks for microbes and drugs. The associated iterative update process has a time complexity of *O*(*m*^3^) + *O*(*n*^3^). For the construction of dual hypergraphs in DHCLHAM, both the KNN algorithm and the KO algorithm are employed. The KNN algorithm, with a time complexity of *O*(*m*^2^) + *O*(*n*^2^), is used to identify the *k* nearest neighbors for each node. The KO algorithm utilizes *K*-means clustering to compute the clustering center vectors, which has a time complexity of *O*(*m*·*c*·*t*) + *O*(*n*·*c*·*t*), where *c* is the number of cluster centers and *t* is the number of iterations. Following this, the strategy update introduces a time complexity of *O*(*N*·*D*), where *N* is the population size and *D* is the dimensionality of the search space. Overall, the KO algorithm has a time complexity of *O*(*m*·*c*·*t*) + *O*(*n*·*c*·*t*) + *O*(*N*·*D*). Summing up the time complexities of all the aforementioned operations, the total time complexity for the first step is *O*(*m*^3^) + *O*(*n*^3^).

In the second step, DHCLHAM's hierarchical attention mechanism is explicitly designed to be computationally efficient by performing attention calculations on local neighborhoods. For Hyperedge-Level Attention, the model first learns to aggregate node information to form hyperedge representations. For each hyperedge e_*i*_ containing *k*_*i*_ = *y*_*i*_ nodes, the attention mechanism calculates weights for all nodes within that hyperedge. This corresponds to a local attention operation with a complexity of *O*(*k*^2^). Subsequently, the model updates each node's representation by attending over its connected hyperedges. For each node *n*_*j*_ connected to d_*j*_ hyperedges, the attention mechanism calculates weights for these neighboring hyperedges. This is another local attention operation with a complexity of *O*(*d*^2^). The total complexity of our hierarchical attention module is therefore *O*(*k*^2^)+*O*(*d*^2^). The model then uses HGCN to learn embeddings for microbes and drugs, a process characterized by a time complexity of *O*(*|E|*·*C*·*F*), where *|E|* signifies the number of hyperedges in HGCN, *C* is the dimensionality of input features, and *F* corresponds to the dimensionality of output features. Additionally, during the contrastive learning phase, DHCLHAM measures similarities among all nodes, adding a time complexity of *O*(*m*^2^)+*O*(*n*^2^). Hence, the aggregate time complexity for this step is *O*(*|E|*·*C*·*F*)+*O*(*m*^2^)+*O*(*n*^2^).

In the third and final step, DHCLHAM integrates the embeddings from the dual hypergraphs using an integrated network primarily that utilizes a multi-head attention mechanism. The time complexity for this integration is *O*(*m*^2^·*h*)+*O*(*n*^2^·*h*), with *h* representing the number of attention heads.

In summary, the total time complexity for training DHCLHAM is the sum of the time complexities from all three steps. After disregarding constant factors from each step, the overall time complexity can be succinctly expressed as *O*(*m*3)+*O(n*3).

Table 2 compares our model with the baseline methods in terms of computational time complexity. In comparison to simpler models such as GCNMDA (Long et al., 2020a), whose per-iteration training complexity is approximately *O*(*n*^3^)+*O*(*n*·*H*·*M*), primarily determined by random walks and the matrix multiplication in the decoder, the time complexity of ordinary GNN models mainly stands at *O*(*|A|*·*F*), predominantly governed by the graph's edges. Our model's per-iteration training complexity is higher, which is chiefly attributed to the quadratic relationship with the number of nodes brought about by the dual-view attention and contrastive learning modules. This represents a deliberate trade-off, where increased computational investment is made to capture more complex relationships and achieve higher prediction accuracy. The dominant cubic complexity *O*(*m*3)+*O*(*n*3) in our framework originates from the one-time similarity fusion preprocessing step, whereas the computational load during the iterative training phase is comparable to other attention-based GNN architectures.

**Table 2:** Compare the time complexity of other methods with ours.

**Method**	**Core algorithm**	**Time complexity**	**MDAD highest AUC**
GCNMDA (Long et al., 2020a)	Graph convolutional network, conditional random field	*O*(*n^3^*)*+O*(*n*·*H*·*M*)	0.9423 ± 0.0105
KNDM (Chen et al., 2025)	Knowledge-graph transformer, semantic feature learning strategy with recursive gating	*O*(*m*^2^)+*O*(*n*^2^)	0.9688 ± 0.0032
HGCLMDA (Hu et al., 2023)	Hypergraph convolutional network, cross-view contrastive learning	*O*(*m*^3^)+*O*(*n*^3^)	0.9762 ± 0.0029
SCSMDA (Tian et al., 2023)	Structure-enhanced contrastive learning, structure-enhanced contrastive learning	*O*(*m*^3^)+*O*(*n*^3^)+*O*(*T·m·n*)	0.9407 ± 0.0044
NRGCNMDA (Du et al., 2025)	Graph convolutional network incorporating a fusion residual network mechanism, conditional random fields	*O*(*m*·*n*·*F*)	0.9516 ± 0.0024
MCHAN (Li et al., 2024)	Contrastive learning, contrastive learning, graph convolutional networks	*O*(*L*·*S*·*F*)+*O*(*m/n*·*F*)	0.9538 ± 0.0068
DHCLHAM	Hypergraphs contrastive learning, non-linear fusion, hierarchical attention mechanisms	*O*(*m*^3^)+*O*(*n*^3^)	0.9827 ± 0.0018

In the column of time complexity, *m* represents the number of microbe nodes, *n* represents the number of drug nodes, *F* indicates the feature dimension of the node, *L* is the number of layers, *H* is the adjacency matrix, and *S* is the sparsity of the adjacency matrix.

### 3.2 Experimental setup and evaluation metrics

A five-fold cross-validation approach is employed to thoroughly evaluate the efficacy of DHCLHAM and the baseline methods on the MDAD, aBiofilm, and DrugVirus datasets. For each dataset, confirmed microbe-drug pairs are positive samples, making up the positive set. Unverified pairs are negative samples, forming the negative set. Then, from the negative set, we randomly pick the same number of samples as the positive set to create the 5-fold cross-validation set. The dataset is divided into five equal subsets, with each subset being successively assigned as the test set, while the other four subsets are utilized for model training. This guarantees that each subset functions as both a training and testing set in various iterations. We then calculate the quantities of true positives (TP), false positives (FP), true negatives (TN), and false negatives (FN) to assess model performance. The assessment metrics comprise the area under the receiver operating characteristic curve (AUC), the area under the precision-recall curve (AUPR), and the F1-score. The metrics are delineated as presented in Equations 18–21.


(18) $$\begin{array}{l} Recall=\frac{TP}{FN+TP} \end{array}$$

(19) $$\begin{array}{l} Precision=\frac{TP}{FP+TP} \end{array}$$

(20) $$\begin{array}{l} Accuracy=\frac{TP+TN}{TP+TN+FP+FN} \end{array}$$

(21) $$\begin{array}{l} F1-score=\frac{2\times Precison\times Recall}{Precison+Recall} \end{array}$$

### 3.3 Performance evaluation

**Figure 4:**
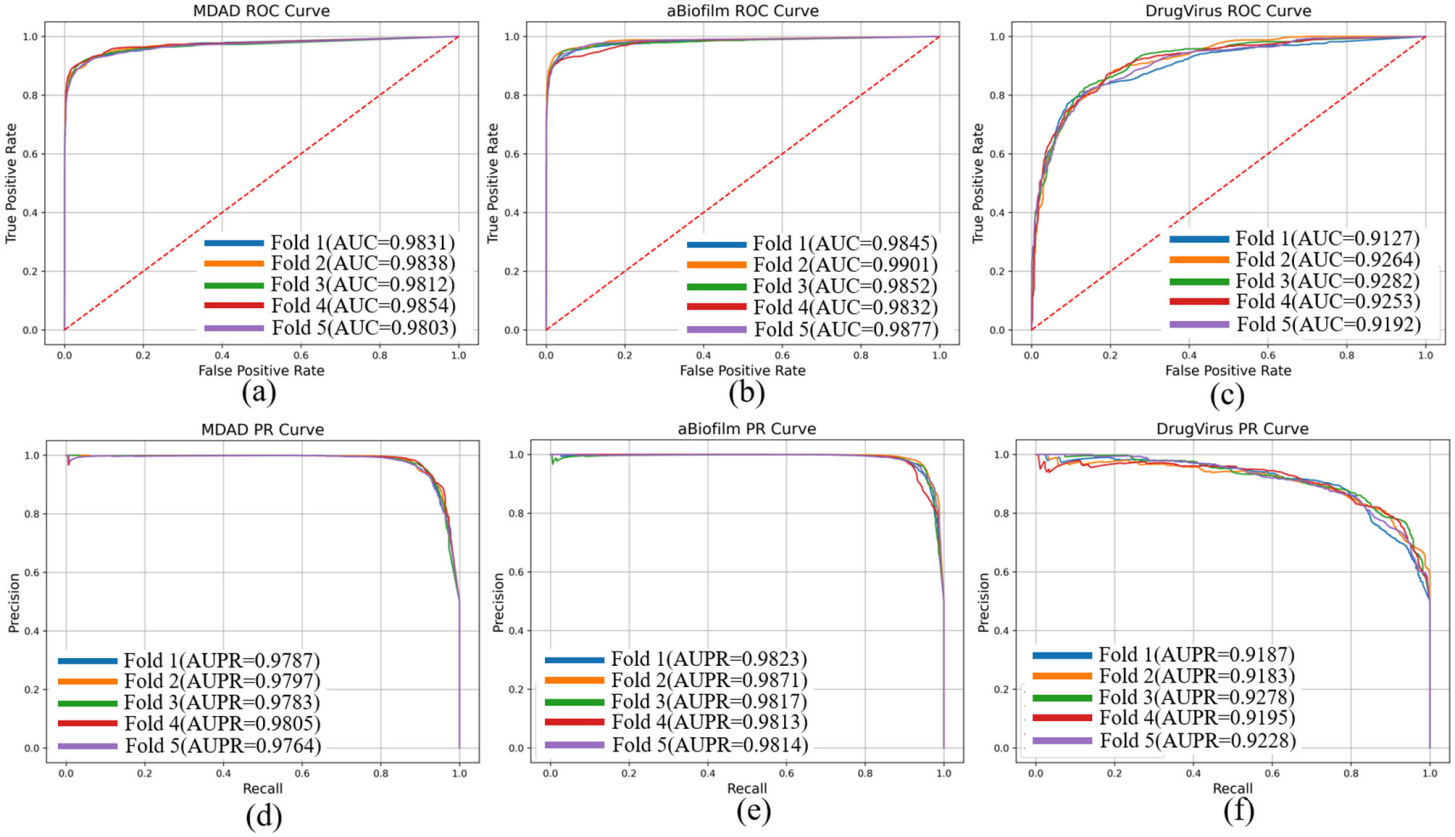
The ROC and PR curves of DHCLHAM for predicting microbe-drug associations on MDAD, aBiofilm and DrugVirus datasets based on 5-fold cross-validation. **(a–f)** Correspond to the ROC and PR results for the three datasets, respectively.

To evaluate the performance of our proposed DHCLHAM model, we conducted 5-fold cross-validation on the MDAD, aBiofilm, and DrugVirus datasets and plotted the AUC and AUPR curves. As shown in Figure 4, the DHCLHAM model exhibited outstanding performance across all three datasets. Specifically, on the MDAD dataset, the model achieved an average AUC of 98.27% and an average AUPR of 97.87%. On the aBiofilm dataset, the average AUC and AUPR values were 98.61% and 98.33%, respectively. Meanwhile, on the DrugVirus dataset, the average AUC was 92.23% and the average AUPR was 92.14%. We also calculated the standard deviations. The standard deviations of AUC and AUPR for the 5-fold cross-validation on the MDAD dataset were 0.0018 and 0.00139, respectively, indicating relatively small fluctuations and stable model performance. For the aBiofilm dataset, the standard deviations were 0.0025 for AUC and 0.00215 for AUPR, showing slightly larger fluctuations and somewhat reduced stability. The DrugVirus dataset, being the smallest in size, had the largest standard deviations of 0.0057 for AUC and 0.0036 for AUPR. Overall, these results demonstrate that the DHCLHAM model can accurately predict microbial responses to different drugs across various datasets.

### 3.4 Baseline models

To demonstrate the superiority of the proposed method, we compared DHCLHAM with six state-of-the-art approaches, including the classic graph-structured GCNMDA (Long et al., 2020a), the latest Graph Transformer-based KNDM (Chen et al., 2025), hypergraph contrast learning-based HGCLMDA (Hu et al., 2023), standard graph contrast learning-based SCSMDA (Tian et al., 2023), the latest microbe-drug association-based NRGCNMDA (Du et al., 2025), and MCHAN (Li et al., 2024).

GCNMDA (Long et al., 2020a): This study is the first to employ a graph structure to represent microbe-drug association data. A heterogeneous network of drugs and microbes is constructed and represented utilizing the random walk with restart (RWR) algorithm. A conditional random field (CRF) layer is incorporated into a graph convolutional network (GCN) framework, featuring an attention mechanism that updates the node embeddings. A bipartite network of microbes and drugs has been reconstructed.

KNDM (Chen et al., 2025): This approach initially constructs a knowledge graph comprising drug and microbe entities to reveal the similarities and associations between entities. Subsequently, an entity category-sensitive Transformer (ECST) is proposed to integrate the diverse entity types and their complex relationships.

HGCLMDA (Hu et al., 2023): This method combines GCN and HGCN to capture local and global structural info from mRNA-drug bipartite graphs, mining high-order relationships between mRNA-drug pairs. It also uses a cross-view contrastive learning architecture to boost learning ability.

SCSMDA (Tian et al., 2023): This approach develops similarity and meta-path induction networks for microorganisms and drugs. It improves node embeddings via a structure-enhanced contrastive learning approach and employs a self-paced negative sampling technique to identify the most informative negative samples, subsequently training an MLP classifier for association prediction.

NRGCNMDA (Du et al., 2025): Shallow features are derived from a microbe-drug heterogeneous network using Node2vec. A Residual Graph Convolutional Network (REGCN) is utilized to capture long-range dependencies through skip connections. A CRF layer imposes contextual constraints to refine the embeddings, and association scores are ultimately computed using a bilinear decoder.

MCHAN (Li et al., 2024): A graph convolutional network incorporating an attention mechanism is employed to extract essential information. Two network topologies are established: a super heterogeneous graph featuring super nodes, and a conventional heterogeneous graph. Graph embeddings are directed through a cross-contrastive learning task, and the outputs of the graph convolutional networks are integrated with an attention mechanism to forecast associations.

**Figure 5:**
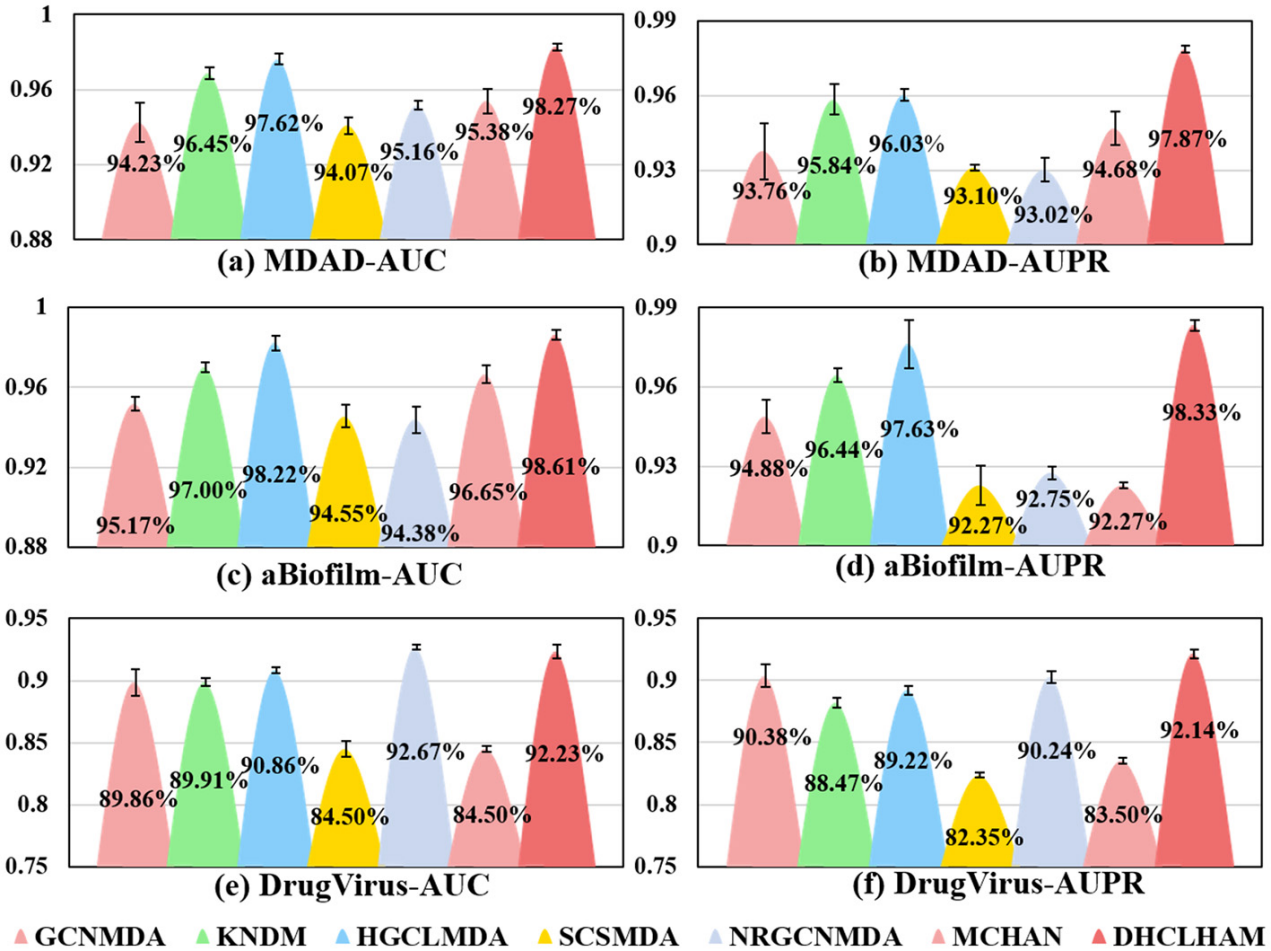
Visual comparisons of AUC and AUPR values for DHCLHAM and six other methods on the MDAD, aBiofilm, and DrugVirus datasets are presented. Different colors denote various methods, with the y-axis representing values of AUC and AUPR. **(a–f)** Correspond to the AUC and AUPR results for the three datasets, respectively.

Figure 5 shows the evaluation of DHCLHAM against baseline methods utilizing AUC and AUPR metrics on the MDAD, aBiofilm, and DrugVirus datasets. The AUC for the DrugVirus dataset is 0.9223, marginally lower than NRGCNMDA's 0.9267. However, DHCLHAM's AUPR of 0.9214 notably surpasses NRGCNMDA's 0.9024, potentially due to the restricted sample size of the DrugVirus dataset. Additionally, on the MDAD and aBiofilm datasets, DHCLHAM attains AUC values of 0.9827 and 0.9861, and AUPR values of 0.9787 and 0.9833, respectively, surpassing all baseline methods. Meanwhile, to verify whether the performance of DHCLHAM is statistically significantly higher than that of other baseline models, we calculated a paired *t*-test of 5-fold cross-validation of DHCLHAM and six other methods. Statistical analysis indicates that DHCLHAM significantly outperforms other methods in these evaluation indicators, with a *p*-value less than 0.05, as shown in Table 3. This enhancement may stem from employing hypergraph structures to encapsulate high-dimensional data and implementing a bi-level hypergraph attention mechanism, alongside contrastive learning across dual hypergraph perspectives. DHCLHAM more effectively models the intricate interactions between microbes and drugs, thereby improving predictive performance.

**Table 3:** Comparison of DHCLHAM with other methods via paired *t*-test on MDAD dataset.

***P*-value**	**GCNMDA**	**KNDM**	**HGCLMDA**	**SCSMDA**	**NRGCNMDA**	**MCHAN**
AUC	3.71e-4	1.64e-5	9.77e-3	2.65e-6	2.33e-8	1.56e-4
AUPR	6.16e-4	6.3e-4	1.24e-5	5.06e-9	5.68e-6	2.49e-3

### 3.5 Ablation experiment

To evaluate the importance of specific modules within the DHCLHAM model, we performed an ablation study concentrating on three essential components across three datasets. This analysis assesses the impact of each module on the overall model efficacy.

#### 3.5.1 Effects of hierarchical attention and contrast learning

NoDHA: This variant eliminates the hierarchical attention mechanism (DHA) and utilizes conventional graph attention instead.

NoCL: In this configuration, the contrastive learning (CL) module for hypergraph contrastive learning is excluded, and the output from the hypergraph convolution is directly input into the integration network for feature amalgamation.

The NoDHA variant exhibits inferior performance relative to DHCLHAM across all four evaluation metrics, AUC, AUPR, F1-score, and ACC, on all three datasets. The decline in performance may arise from the scarcity of microbial-drug interaction data, as the bi-level hierarchical attention mechanism facilitates the acquisition of higher-order information, thus enhancing the network representation. The introduction of hierarchical attention updates node embeddings at the hyperedge level, thereby enabling iterative refinement of node information. This highlights the essential function of the hierarchical attention mechanism.

**Figure 6:**
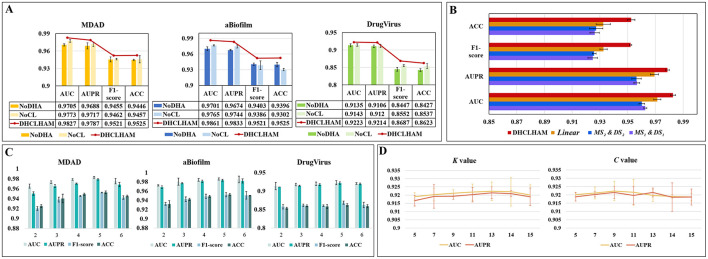
**(A)** Ablation experiments of DHCLHAM with different metrics compared under the two modules NoDHAL and NoCLTF. **(B)** Impact of non-linear fusion in the MDAD dataset. **(C)** The influence of DHCLHAM on predictive performance across varying head counts in three datasets. **(D)** Impact of DHCLHAM on predicting performance at different values of *K* and *C* on DrugVirus.

The elimination of the contrastive learning module (NoCL) leads to a significant decrease in all four metrics, indicating that hypergraph contrastive learning plays a crucial role in differentiating and augmenting node information from various perspectives. This consequently enhances predictive accuracy. These findings collectively affirm the essentiality of each component within DHCLHAM and validate the superior efficacy of the fully integrated model. The findings are depicted in Figure 6A.

#### 3.5.2 Effects of non-linear fusion

Non-linear fusion can inherently incorporate diverse similarity information from both microbial and drug feature domains, thereby significantly enhancing the model's predictive capability. The efficacy of non-linear fusion was evaluated by executing the model with a singular similarity and a linear fusion of two similarities. Specifically:

*MS*_1_&*DS*_1_: In the data preprocessing phase, only microbial functional similarity and drug structural similarity were used.

*MS*_2_&*DS*_2_: In the data preprocessing phase, only microbial Gaussian kernel similarity and drug Gaussian kernel similarity were used.

*Linear*: During the data preprocessing phase, linear average fusion was employed for microbial functional similarity and microbial Gaussian kernel similarity, as well as for drug structure similarity and drug Gaussian kernel similarity.

Our proposed model, employing the previously mentioned similarity combinations, was assessed on the MDAD dataset using identical parameter configurations. As illustrated in Figure 6B, t the performance of *MS*_1_&*DS*_1_, *MS*_2_&*DS*_2_, and *Linear* on the MDAD dataset deteriorated. This indicates that non-linear fusion effectively captures the non-linear relationships between microbial and drug similarity perspectives, thereby improving overall model performance.

### 3.6 Parametric analysis

Multiple critical parameters influence the efficacy of the DHCLHAM model. This section addresses four essential hyperparameters: the quantity of attention heads (head), the count of HGCL layers, the value of *K* in the KNN method for hypergraph construction, and the number of clustering centers *C* in the KO method. Relevant experiments were performed, and the outcomes were assessed utilizing AUC, AUPR, F1-score, and ACC as performance metrics.

(1) The effect of altering the number of multi-head attention heads is depicted in Figure 6C, where the head value is chosen from the set {2, 3, 4, 5, 6}. The MDAD and aBiofilm datasets exhibit optimal performance across evaluation metrics when the head count is configured to 5. Conversely, for the DrugVirus dataset, optimal metric values are achieved with a head count of 4.
(2) The impact of the number of HGCL layers is encapsulated in Table 4, to avoid overfitting, the number of HGCN layers is set to {1, 2, 3, 4}. The optimal values, highlighted in bold in the table, indicate that the model attains maximum performance across all three datasets when employing two layers.
(3) The impact of the *K* value in the KNN method and the number of cluster centers *C* in the KO method on hypergraph construction is also analyzed. *K* and *C* ascertain both the length and quantity of the hyperedges, rendering their selection crucial. Utilizing the DrugVirus dataset as a reference, Figure 6D illustrates that DHCLHAM attains peak performance with *K* configured at 13 and *C* configured at 9.

**Table 4:** Impact of DHCLHAM in predicting performance under different number of HGCN layers on three datasets.

**Dataset**	**Number of HGCN**	**AUC**	**AUPR**	**F1-score**	**ACC**
MDAD	1	0.9793	0.9735	0.9294	0.9309
	2	**0.9827**	**0.9787**	**0.9521**	**0.9525**
	3	0.9802	0.9746	0.9365	0.9355
	4	0.9693	0.9604	0.9180	0.9196
aBiofilm	1	0.9822	0.9769	0.9487	0.9457
	2	**0.9861**	**0.9833**	**0.9521**	**0.9525**
	3	0.9834	0.9802	0.9488	0.9483
	4	0.9723	0.9664	0.9377	0.9377
DrugVirus	1	0.9184	0.9178	0.8588	0.8545
	2	**0.9223**	**0.9214**	**0.8687**	**0.8623**
	3	0.9156	0.9113	0.8511	0.8503
	4	0.9083	0.9042	0.8330	0.8426

Values in bold denote the best performance achieved for each metric.

### 3.7 Case studies

To thoroughly evaluate the efficacy of DHCLHAM in discovering novel microbe-drug associations (MDAs) and in analyzing the interpretability of its predictions, two case studies were formulated: one focused on literature validation and the other employing network pharmacology analysis.

#### 3.7.1 Case 1

**Figure 7:**
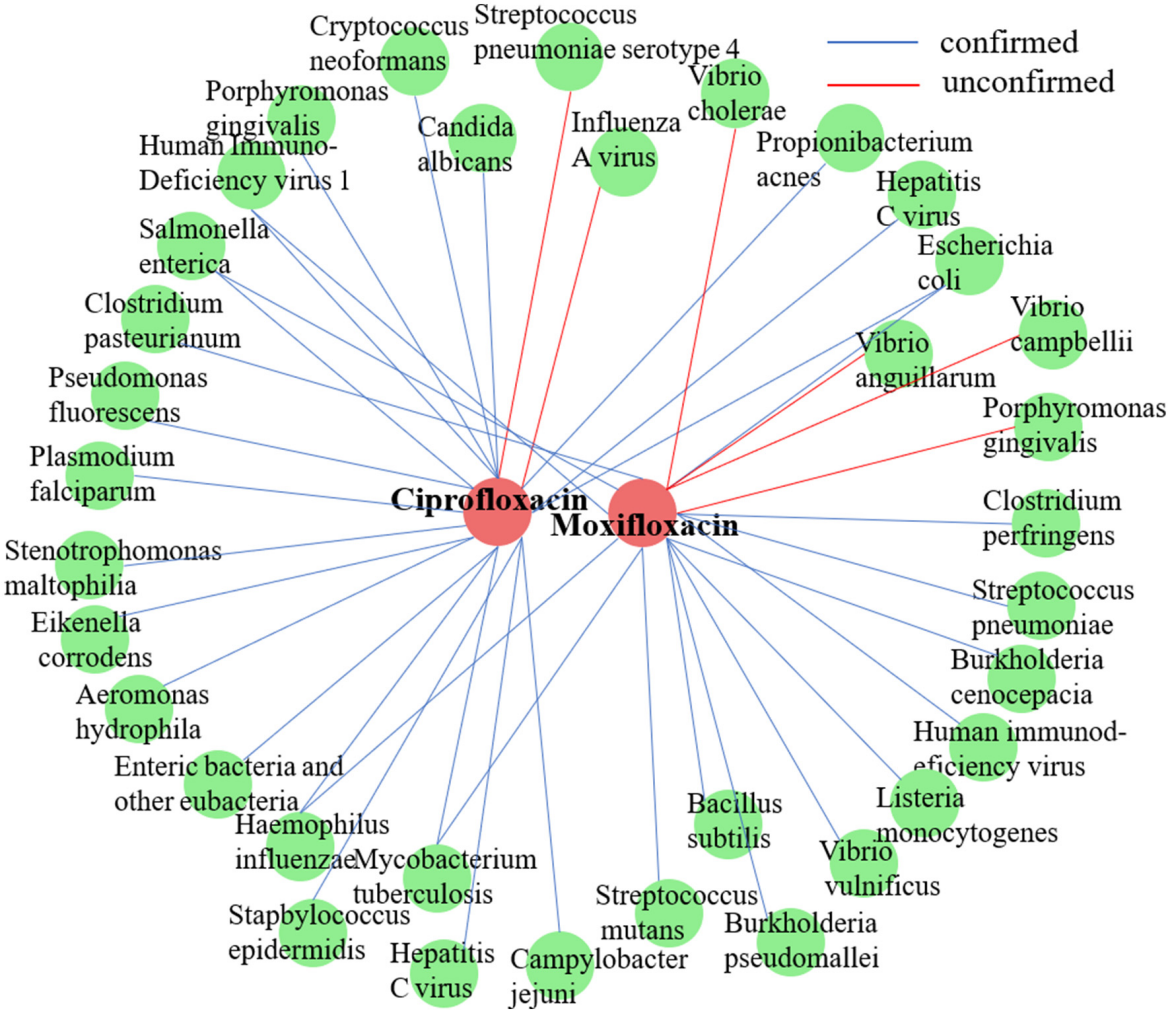
Validation results of the top 20 microbes predicted by DHCLHAM to be associated with two drugs (ciprofloxacin and moxifloxacin) are shown. Blue lines indicate validated associations, while red lines represent unvalidated ones. Red indicates drugs, and green indicates microbes.

In accordance with the methodology of a prior study (Long et al., 2020a), we conducted a case study on two commonly employed antimicrobial agents, ciprofloxacin and moxifloxacin, using the MDAD dataset. For each target drug, all established microbe-drug associations were regarded as unknown. Next, DHCLHAM was utilized to rank all candidate microbes in descending order based on their predicted association scores. The highest 20 ranked microbes for each drug were subsequently chosen and corroborated with existing literature. Figure 7 visualizes the anticipated microbial associations for both ciprofloxacin and moxifloxacin.

Ciprofloxacin, a second-generation fluoroquinolone, is frequently prescribed for respiratory tract infections and sepsis. It demonstrates extensive antibacterial efficacy, especially against gram-negative pathogens (Long and Luo, 2021). (Devos et al. 2017) examined the effect of ciprofloxacin on membrane vesicle (MV) secretion in Stenotrophomonas malt philia. Their findings indicated that ciprofloxacin not only stimulated the production of classical outer membrane vesicles (OMVs) but also triggered the formation of outer-inner membrane vesicles (OIMVs), which encapsulate both outer and inner membranes and are enriched in cytoplasmic proteins. Moreover, these OIMVs exhibit trichome-like surface structures and are linked to the release of bacteriophage particles and prophage induction, potentially leading to detrimental effects associated with antibiotic treatment. (Szczuka et al. 2017) demonstrated that ciprofloxacin displayed antibacterial activity against Staphylococcus epidermidis at standard concentrations, although its efficacy was reduced against partially drug-resistant strains. Furthermore, subinhibitory concentrations of ciprofloxacin differentially influenced biofilm-associated gene expression and biofilm morphology in *Stapbylococcus epidermidis* contingent upon the strain. Table 5 presents that of the top 20 microbes anticipated to associate with ciprofloxacin, 18 have been substantiated by current literature, yielding a validation accuracy of 90%. Beyond confirming known associations, our model's unconfirmed predictions may point toward new research avenues. For instance, the high-scoring prediction for Streptococcus pneumoniae serotype 4 suggests a testable biological hypothesis: Ciprofloxacin may possess notable efficacy against this specific serotype. This provides a clear direction for experimental validation and could potentially inform drug development strategies by refining treatment guidelines for infections caused by this pathogen.

**Table 5:** Top 20 predicted ciprofloxacin-associated microbes.

**Microbe**	**Evidence**	**Microbe**	**Evidence**
*Escherichia coli*	PMID:26607324	Enteric bacteria and other eubacteria	PMID:36682905
*Stenotrophomonas maltophilia*	PMID:28488744	Human immunodeficiency virus 1	PMID: 9566552
*Haemophilus influenzae*	MDAD	*Propionibacterium acnes*	MDAD
*Mycobacterium tuberculosis*	PMID:30020039	Influenza A virus	Unconfirmed
*Porphyromonas gingivalis*	PMID:26369485	*Salmonella enterica*	PMID:26933017
*Eikenella corrodens*	PMID:16875802	*Pseudomonas fluorescens*	PMID:30026133
*Candida albicans*	PMID:31471074	*Aeromonas hydrophila*	PMID:24242249
*Stapbylococcus epidermidis*	PMID:28481197	Hepatitis C virus	PMID:12234860
*Campylobacter jejuni*	PMID:27900889	*Streptococcus pneumoniae* serotype 4	Unconfirmed
*Plasmodium falciparum*	PMID:17214980	*Cryptococcus neoformans*	PMID:29858266

Moxifloxacin is a broad-spectrum antimicrobial agent categorized as a fourth-generation quinolone antibiotic. It is commonly prescribed for the treatment of upper and lower respiratory tract infections, such as acute sinusitis, pneumonia, and infections of the skin and soft tissues. An experimental study by (Chon et al. 2018) revealed that Clostridium perfringens exhibited significant susceptibility to moxifloxacin, thereby substantiating its clinical efficacy in treating infections caused by this pathogen and underscoring its potential as a viable therapeutic option. Furthermore, (Butt et al. 2014) indicated that the overexpression of the HicA toxin in *Burkholderia pseudomallei* led to bacterial growth inhibition and a heightened population of persister cells resistant to ciprofloxacin or ceftazidime, thereby emphasizing the potential of moxifloxacin as an effective antibacterial agent in particular circumstances. However, not all pathogenic bacteria demonstrate significant sensitivity to moxifloxacin. Table 6 indicates that of the top 20 microbes anticipated to associate with moxifloxacin, 16 have been substantiated by current literature, yielding a validation accuracy of 80%. Among the four unconfirmed candidates in the top-20 list, *Vibrio cholerae*, the causative agent of cholera, was identified with a high association score. This leads to a clinically significant and actionable hypothesis: Moxifloxacin may be an effective antibacterial agent against *Vibrio cholerae*. This is a plausible hypothesis, as moxifloxacin is a broad-spectrum fluoroquinolone known to target bacterial DNA replication. This prediction can be directly validated through *in vitro* antimicrobial susceptibility testing. Given the growing challenge of antibiotic resistance in *Vibrio cholerae*, this finding could inform drug development strategies by highlighting moxifloxacin as a potential candidate for treating cholera, particularly in cases resistant to standard therapies.

**Table 6:** Top 20 predicted moxifloxacin-associated microbes.

**Microbe**	**Evidence**	**Microbe**	**Evidence**
*Candida albicans*	PMID: 12121916	*Vibrio anguillarum*	Unconfirmed
Human immunodeficiency virus 1	PMID:18441333	*Escherichia coli*	PMID:31542319
*Burkholderia pseudomallei*	PMID:24502667	*Vibrio campbellii*	Unconfrmed
*Haemophilus influenzae*	PMID: 11856249	*Vibrio vulnificus*	PMID:10632381
*Salmonella enterica*	PMID:22151215	*Bacillus subtilis*	PMID:30036828
*Clostridium perfringens*	PMID:29486533	*Streptococcus mutans*	PMID:29160117
Human immunodeficiency virus	PMID:18441333	*Porphyromonas gingivalis*	Unconfirmed
*Mycobacterium tuberculosis*	PMID:35975988	*Streptococcus pneumoniae*	PMID:31542319
*Vibrio cholerae*	Unconfirmed	*Burkholderia cenocepacia*	PMID:28355096
*Listeria monocytogenes*	PMID:28739228	*Clostridium pasteurianum*	PMID:29486533

#### 3.7.2 Case 2

**Figure 8:**
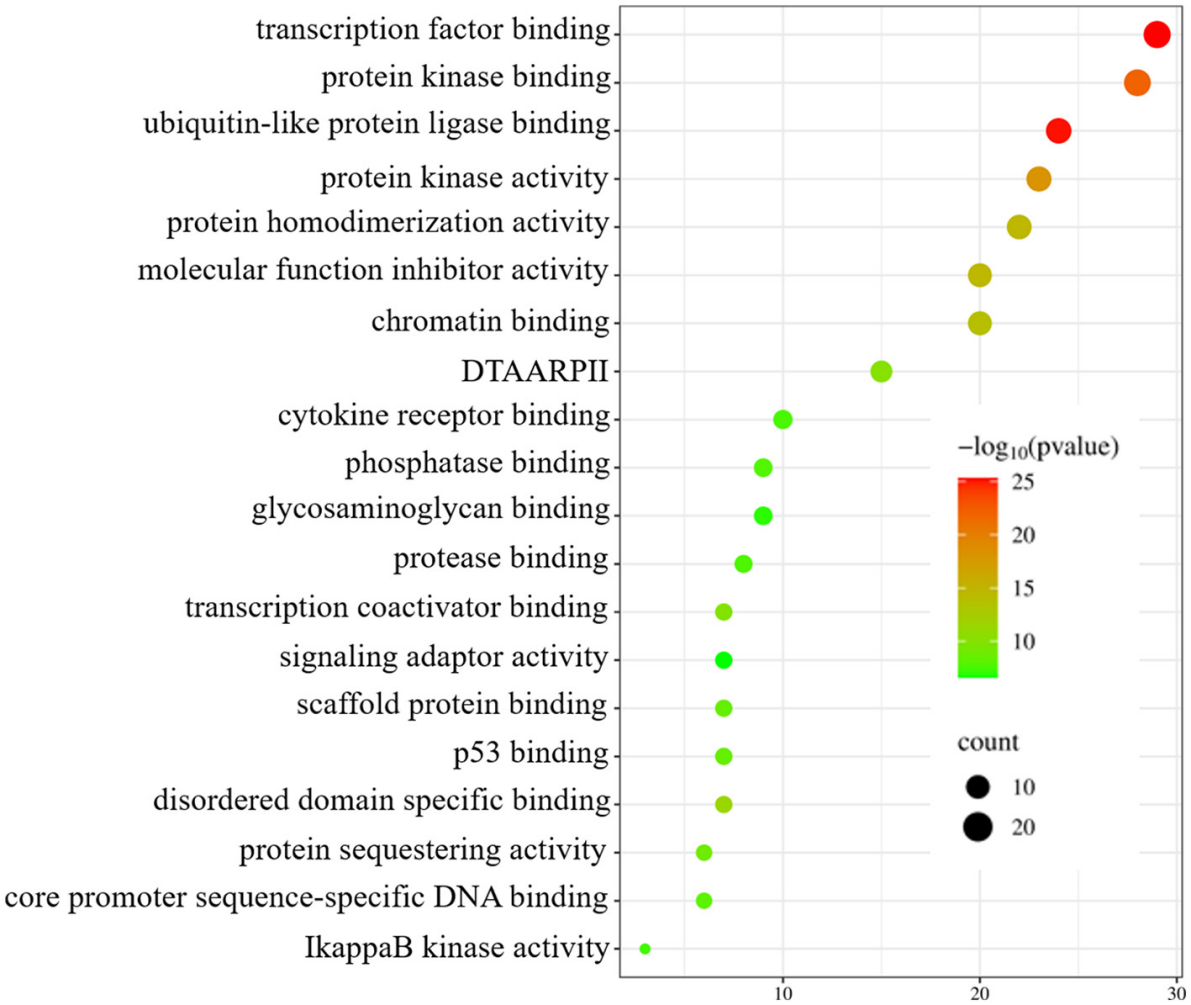
Enrichment analysis bubble diagram. On the left side are specific biological processes (DTAARPII represent DNA-binding transcription activator activity and RNA polymerase II-specific).

**Figure 9:**
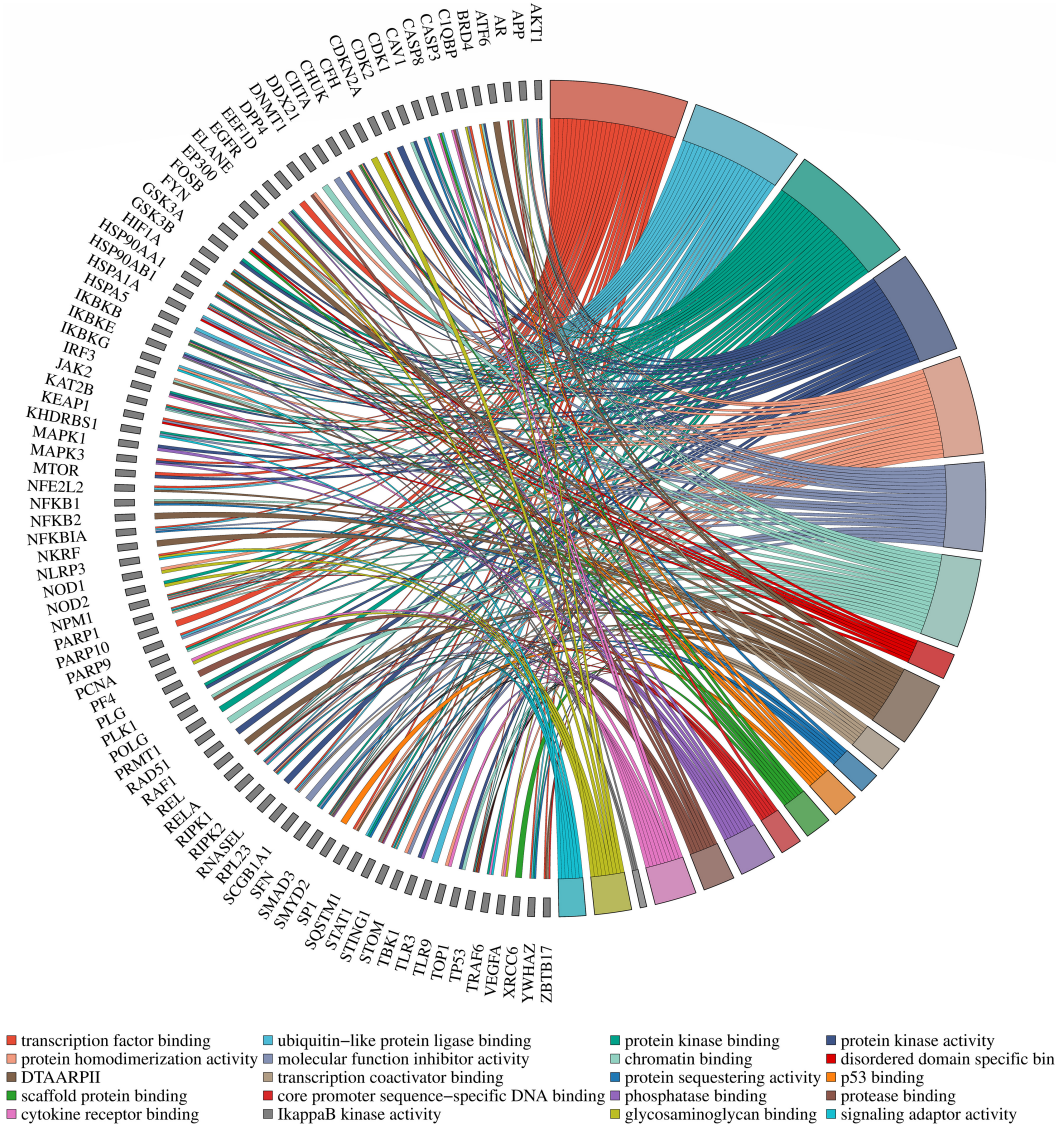
Diagram of gene pathway correlations. Gene names are located on the left periphery, while specific biological processes are positioned on the right. Connecting lines denote the relationship between each gene and its corresponding process.

To enhance the validation of the model's interpretability in predicting microbe-drug associations, we employed a network pharmacology approach to analyze the SARS-CoV-2 virus as a case study. The model's prediction scores were employed to identify the twenty drugs most pertinent to the SARS-CoV-2 virus. Fourteen drugs were validated through a literature review, as shown in Table 7. Secondly, we acquired the genetic information of the SARS-CoV-2 virus and the target data for 20 validated and unvalidated drugs from four databases: GeneCards, NCBI, UniProt, and OMIM, to guarantee thorough gene representation. In the GeneCards database, we selected genes with scores exceeding the median of the “Score” column to acquire more precise gene information. We used the “Human” tag in the NCBI, UniProt, and OMIM databases to obtain specific gene information. The gene data from these four databases was amalgamated and deduplicated to derive the final gene set for the SARS-CoV-2 virus. We acquired target protein information for the drugs from the ChEMBL database and used UniProt to correlate these proteins with human genes. For compounds lacking target information in ChEMBL, we used the SwissTargetPrediction database to forecast targets. Upon acquiring the target information for all validated and unvalidated drugs, we discovered that merely two target genes were exclusive to the unvalidated drugs. Consequently, we omitted these two genes from the target gene set and eliminated duplicates from the aggregated gene set. Ultimately, we employed the SARS-CoV-2 viral genes and the drug target genes for Gene Ontology enrichment analysis. Figure 8 presents a bubble diagram displaying the enrichment outcomes for designated pathways. The pathway with the highest enrichment is transcription factor binding, whereas the pathway with the lowest enrichment is IkappaB kinase activity. In the protein kinase binding pathway, host cells infected by SARS-CoV-2 exploit host protein kinases to promote viral replication and transcription (Huang et al., 2022). SARS-CoV-2 infection activates or disrupts host transcription factors, thereby eliciting immune and other responses. The virus depends on host transcription factors for its replication and transcription, influencing their recruitment. This pathway is essential for immune cell functionality and infection-related pathology (Gordon et al., 2020). Figure 9 shows the enrichment of specific genes across these 20 biological processes. SARS-CoV-2 infection exploits or disrupts the binding mechanism of ubiquitin-like protein ligases in host cells within the ubiquitin-like protein ligase binding pathway (Gonzalez-Orozco et al., 2024). Clearly, enrichment analysis effectively validates and elucidates the model's predictions regarding microbe-drug associations.

**Table 7:** Top 20 predicted SARS-CoV-2 related drugs.

**Drug**	**Evidence**	**Drug**	**Evidence**
Chlorpromazine	PMID:33387629	Nitazoxanide	PMID:36066651
Brequinar	PMID:36041646	Ritonavir	PMID:35183067
Favipiravir	PMID:33108587	N-MCT	Unconfirmed
Leflunomide	PMID:37534317	Filociclovir	Unconfirmed
Foscarnet	PMID:34638812	Ganciclovir	Unconfirmed
Fosamprenavir	Unconfirmed	Brincidofovir	PMID:32834922
Acetylsalicylic acid	PMID:36298484	Letermovir	PMID:33970450
Arbidol (Umifenovir)	PMID:32955901	Labyrinthopeptin A1	Unconfirmed
Lamivudine	Unconfirmed	Camostat	PMID:33176395
Uracil	PMID:35337173	Emetine	PMID:33302852

## 4 Conclusion and discussion

This study presents a dual hypergraph contrastive learning model that integrates a hierarchical attention mechanism to derive initial features for predicting microbe—drug interactions. The model creates two hypergraph structures by combining the original bipartite graph with a similarity matrix non-linear fused using KNN and KO algorithms. The resultant hyperedges facilitate the modeling of intricate relationships among various drugs and microbes from multiple viewpoints. Unlike traditional graph attention mechanisms, the proposed hierarchical attention mechanism systematically aggregates information at both hyperedge and node levels within the hypergraph. In contrast to traditional graph-based contrastive learning methods, we conduct contrastive learning on both KNN-based and KO-based hypergraphs. We subsequently integrate data from these two hypergraphs using a multi-head attention mechanism to produce the final embedding representations of microbes and drugs, which are subsequently employed to calculate microbe-drug association scores. Assessment across various metrics on multiple publicly accessible datasets indicates that the DHCLHAM model surpasses six leading baseline models. Finally, we substantiate the predictive efficacy of DHCLHAM in identifying novel microbe-drug interactions via literature validation and case studies in network pharmacology.

However, the DHCLHAM model demonstrates specific limitations, primarily attributable to the inadequate compilation of similarity data for drugs and microorganisms. In future endeavors, we plan to investigate supplementary drug-related data sources, encompassing molecular fingerprints, drug affinity profiles, SMILES representations and drug-Target information (Tanvir et al., 2024). We intend to integrate microbial data, including 16S rRNA gene sequences (Wang et al., 2025). The existing methodology creates two varieties of hypergraph structures, yet it may inadequately encompass the complete intricacy of the data. Subsequent research will examine the amalgamation of both statically configured and dynamically developing hypergraph structures (Bindels et al., 2025). Meanwhile, biological experiments will also be considered in the future to verify the prediction rate of the model. Subsequent improvements will concentrate on using supplementary datasets and sophisticated machine learning methodologies to enhance the predictive precision of microbe-drug interactions. Additionally, performing comprehensive case studies on microbe-drug interactions, augmented by network pharmacology analyses, will provide enhanced understanding of the fundamental biological connections between microbes and drugs.

## Data Availability

The original contributions presented in the study are included in the article/Supplementary material. All the code and data related to this paper have been made publicly available here: https://github.com/HZUNie/DHCLHAM/tree/master.
